# An Erect Gait

**Published:** 1870-04

**Authors:** 


### AN ERECT GAIT

Gives to a woman a queenly appearance, and to men an air of manliness, integrity, and fearlessness. To bend forward or downward while walking, indicates debility, depression, or mental trouble, and always aggravates itself and promotes disease. Pads and supporters are all pernicious, are worse than useless, because they teach the system to rely on them, and cannot support one part of the body without causing an unnatural strain on some other part, and, to that extent, tend to disease that part.

There is always one easily available and successful method of acquiring an erect, manly gait, without any material effort, or feeling of awkwardness. Let the chin be a little above a horizontal line, which is easily done by keeping the eye fixed on the top of some person's hat or bonnet in front of you.

The habit of this erect carriage may be facilitated by accustoming yourself, when at home, in the garden, or other places, to walk with the hands behind, held in one another, and the head thrown up, as is done in smoking a cigar or singing a tune.

---

By the courtesy of Professor Henry, of the Smithsonian Institution, we have received the "Annual Report of the Board of Regents," 473 pp. 8vo. It contains a vast amount of philosophical information, full of interest to every general reader, and to all educated men.

